# Autoimmune Pericarditis: Diagnosis and New Therapeutics

**DOI:** 10.1007/s11886-025-02248-1

**Published:** 2025-07-15

**Authors:** Heba Wassif, Elizabeth Ghandakly, Habib Layoun, Jaideep Singh Bhalla, Emily Littlejohn, Tom Kai Ming Wang, Elaine Husni, Allan Klein

**Affiliations:** 1 Department of Cardiovascular Medicine, Cleveland Clinic Foundation, 9500 Euclid Avenue, Cleveland, OH 44195 USA; 2 Department of Internal Medicine, Cleveland Clinic, Cleveland, OH USA; 3 Department of Rheumatology, Cleveland Clinic, Cleveland, OH USA

**Keywords:** Autoimmune pericarditis, Pericarditis, Autoinflammatory

## Abstract

**Purpose of Review:**

To provide an overview of the diagnosis and updated therapeutic approach to autoimmune pericarditis.

**Recent Findings:**

Subtypes of autoimmune pericarditis may benefit from IL-1 inhibition and future phenotyping based on clinical presentation, biomarkers, and imaging may aid in guiding future therapeutic approaches.

**Summary:**

Autoimmune pericarditis is a unique phenotype of pericarditis for which there is scarce data. The clinical spectrum of pericardial involvement associated with autoimmune disorders varies in acuity, recurrence, severity, and symptomatic presentation. There is a pathophysiologic distinction that separates autoimmune and autoinflammatory pericarditis from other pericarditis presentations. This review explores the state of the art in diagnosis and therapeutic management.

## Introduction

Pericarditis is a major cardiac manifestation associated with autoimmune disorders. In 1882, Charcot described pericardial disease in patients with rheumatoid arthritis. Moreover, pericardial involvement extends to other auto-immune disorders such as systemic lupus erythematosus (SLE), Sjogren’s syndrome, systemic sclerosis, and vasculitis, in addition to organ-specific diseases such as inflammatory bowel disease [1–3]. The clinical spectrum of pericardial involvement in autoimmune disorders includes acute, recurrent, incessant, chronic pericarditis and constrictive pericarditis, asymptomatic pericardial effusion, and rarely cardiac tamponade [4]. In some cases, pericardial symptoms herald the presentation of disease, in the absence of other systemic manifestations. Over the past decade, there has been a resurgence of interest in further understanding pericardial disease secondary to autoimmune conditions. There is a clear distinction between the pathophysiology of autoinflammatory and autoimmune conditions, which is reflective of the presentation and prognosis. The aim of this review is to explore the state-of-the-art diagnostics and therapeutics in the management of autoimmune pericarditis (AAP).

### Epidemiology

Acute pericarditis regardless of the etiology represents 5% of patients presenting to the Emergency Department with chest pain. However, the true incidence of pericarditis varies based on the population. The annualized prevalence estimate is 48 patients per 100,000 persons in the U.S [5], compared to a reported estimate of 27.7 cases per 100,000 person years in an urban area in Northern Italy [6]. The overall incidence rate of acute pericarditis increases with age; the ages of patients with acute pericarditis in clinical series have ranged from 33 to 73 years, with a median age of 56 years [7, 8]. The role of gender in acute pericarditis has varied between murine experimental studies and clinical studies with a male/female ratio of 1.42 in some studies [9, 10]. A rheumatic etiology is present in 2–7% of patients with acute pericarditis and around 10% of recurrent pericarditis. Rheumatologic diseases manifesting in pericarditis have been classified immunologically as autoimmune and autoinflammatory.

The following review will focus on autoimmune conditions that are commonly associated with pericarditis, such as systemic lupus erythematosus (SLE), rheumatoid arthritis (RA), systemic sclerosis, Sjogren’s syndrome and vasculitis. The clinical diagnosis of pericarditis is largely based on symptoms. However, the true incidence may be underreported due to asymptomatic cases. For instance, asymptomatic pericardial disease has been identified in more than 50% of SLE patients [11]. Furthermore, the reported rising prevalence of both clinical and subclinical autoimmune conditions may potentially reflect an increase in pericardial conditions [12].

### Pathophysiology

**Fig. 1:**
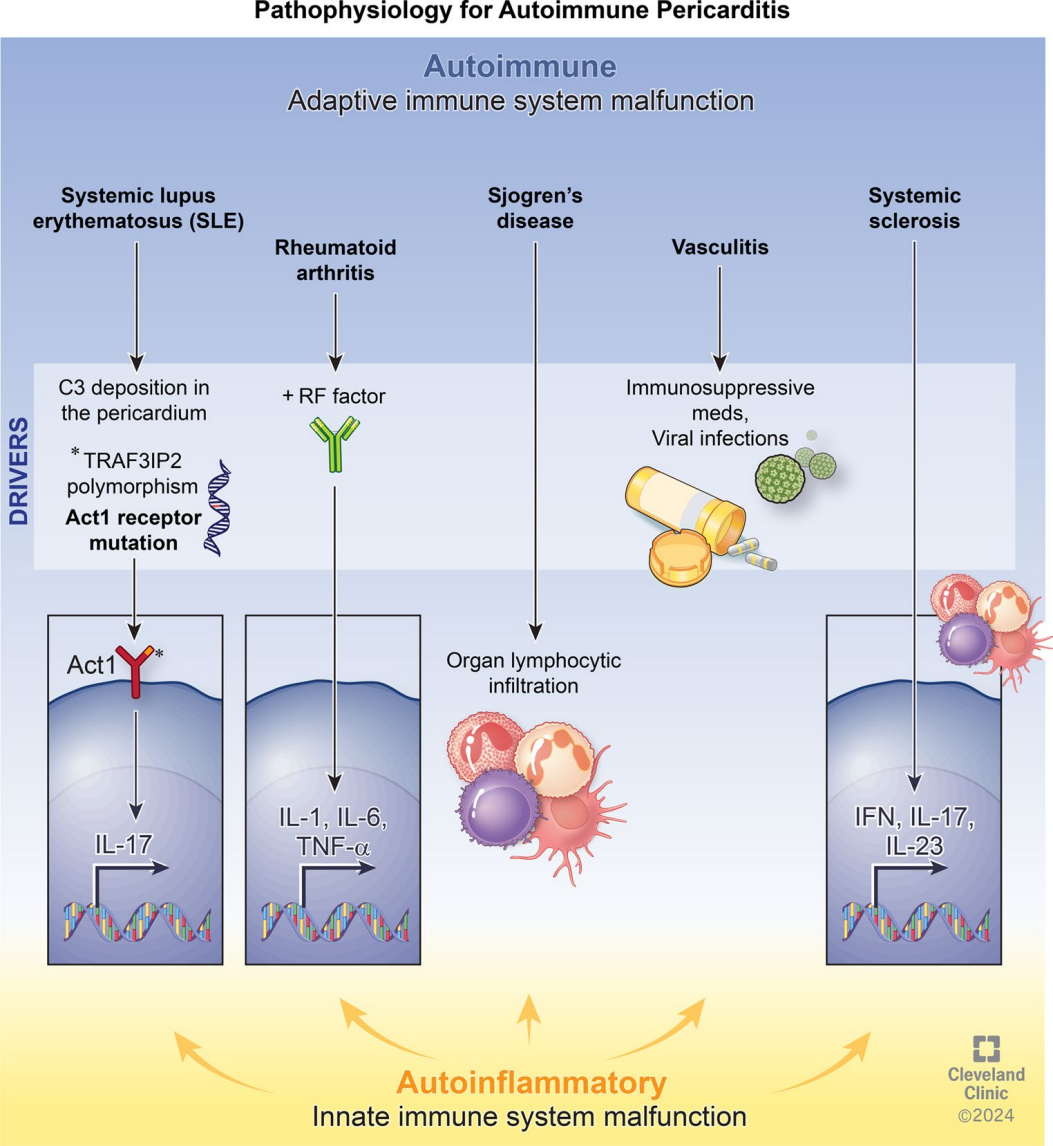
Pathophysiology for autoimmune pericarditis. The *asterisk* (*) signifies the mutation which is also shown on the Act1 receptor with the little *orange* piece. Act1: Act1 adaptor protein, essential intermediate for IL-17; C3: complement component 3; IFN: interferon; IL: interleukin; TNF: tumor necrosis factor; TRAF3IP2: TRAF3 interacting protein 2

Acute pericarditis is a heterogeneous condition characterized by inflammation of the pericardium. The pathophysiology involves an inflammatory response, typically mediated by the innate immune system, with neutrophils and macrophages playing significant roles. Some authors have classified acute pericarditis into two distinct sub types: autoimmune and autoinflammatory. The extent of involvement of the innate and adaptive immunity is based on the subtype. Interleukin-1 and NACHT, leucine-rich repeat, and pyrin domain-containing protein (NLRP3) play a central role in idiopathic recurrent pericarditis and autoinflammatory conditions, while in autoimmune disease such as SLE, there is a predominance of type 1 interferon signature [13, 14]. Even so, not all patients with SLE have the interferon signature [15] (Fig. 1; Table 1).

**Table 1:** Comparison of pathophysiology and presentation of autoimmune diseases associated with pericarditis

Autoimmune Disease	Pericardial Involvement	Pericardial Involvement
Systemic Lupus Erythematosus	Autoantibodies, immune complexes, & complement proteins deposited into pericardial vessel walls and mononuclear cell infiltration.	Asymptomatic > symptomatic > tamponade > constrictive
Rheumatoid Arthritis	IL-1, IL-6, TNF-α upregulation	Asymptomatic effusions > acute > constrictive
Systemic Sclerosis	Endothelial cell dysfunction and fibrosis leading to microvascular disturbances	Autopsy Evidence > Asymptomatic effusions > acute > symptomatic effusions > tamponade > constrictive
Vasculitis	Inflammation of pericardial walls (cell or immune complex mediated)	Acute (Kawasaki > Eosinophilic Granulomatosis with Polyangiitis > Granulomatosis with Polyangiitis)
Sjogren’s Syndrome	Not well-defined; likely cytokine (IFN, IL17/IL23) and B cell infiltration.	Asymptomatic > effusions > constrictive pericarditis

Similar to acute pericarditis, the pathophysiology of recurrent pericarditis (RP) is complex and involves both autoimmune and autoinflammatory mechanisms. Autoimmune contributions are suggested by the presence of autoantibodies and associations with rheumatologic disorders, such as SLE. Autoinflammatory mechanisms are primarily driven by the activation of the inflammasome, particularly the NLRP3 inflammasome, leading to the production of interleukin-1 (IL-1) and similarities with autoinflammatory condition such as Familial Mediterranean Fever (FMF) [16]. The degree of predominance of either mechanism led to the suggestion by Tombettti et al. to stratify patients with recurrent pericarditis into three phenotypes [17]:

a) An **autoimmune** type, highlighted by features occurring in systemic autoimmune diseases (e.g., arthralgias, dry eyes, Raynaud’s phenomenon), moderate C-reactive protein (CRP) elevation, frequent autoantibody positivity (anti-nuclear antibodies (ANA), anti-heart antibodies (AHA) and anti-intercalated disk autoantibodies (AIDA). This subtype shares the hallmarks seen in autoimmune disorders. Anti-nuclear antibodies are a frequent finding in patients having RA, with a prevalence up to 45% compared to 15% of healthy patients [18]. Anti-heart autoantibodies and anti-intercalated disk autoantibodies are present in about 67.5% of patients with RP [19]. The production of such autoantibodies may be due to the above-mentioned processes, where the exposition of autoantigens could stimulate an immune response and the activation of T and B lymphocytes. Alternatively, these autoantibodies may simply be biomarkers without a pathogenic role [20]. This is likely the dominant phenotype in autoimmune disorders, although overlap is present.

b) An **autoinflammatory** phenotype, highlighted by recurrent relapses followed by complete resolution, symptomatic serositis, high fever associated with high CRP and absence of autoantibodies, with major clinical similarities to autoinflammatory disease such as Familial Mediterranean Fever (FMF), or tumor necrosis factor receptor-associated periodic syndrome (TRAPS). It has been suggested that some of these patients might have an atypical or subclinical form of an autoinflammatory disease, e.g., genetic disorders characterized by primary dysfunction of the innate immune system and caused by mutations of genes involved in the inflammatory response.

c) A **nonspecific** phenotype, characterized by mildly symptomatic patients with few attacks, subacute course, smoldering elevation of inflammatory markers, and no evidence of autoimmunity [17].

It is not fully understood how much innate and adaptive immunity contributes to the immunopathogenesis of pericarditis in the setting of autoimmune disease; limited research exists exploring pathophysiological mechanisms that drive the heterogeneity of outcomes observed in specific forms [16, 21]. In the following section, the pathophysiology of pericarditis will be addressed according to specific auto-immune diseases that are the most known to be associated with pericarditis:

### Systemic Lupus Erythematous (SLE)

Acute or chronic inflammation of the pericardium in SLE are mostly thought to be triggered by C3 deposition in the pericardial tissue [22]. Genetic variability coding for innate and adaptive immunity affects the disease phenotype and the risk of pericarditis. A study by Perricone et al. concluded that single nucleotide polymorphism in the *TRAF3IP2* gene that codes for Act1 (a signaling receptor in IL-17 mediated immune processes) was associated with higher risk for pericarditis in SLE patients [23].

### Rheumatoid Arthritis

Although scant literature exists on the pathophysiology of pericarditis in RA, it is usually present in patients with a positive rheumatoid factor (RF). Furthermore, it has been shown that cytokines involved in the inflammatory process of RA (IL-1, IL-6, TNF-α) are also involved in cardiac manifestations of RA including pericarditis [24].

### Systemic Sclerosis

Fluid analyses from pericardial samples in patients with scleroderma were found to be predominantly exudative. In contrast to other auto-immune diseases, there was lack of auto-immune activity, such as autoantibodies, immune complexes and complement deposits [25, 26]. Lefèvre et al. reported a prevalence of 30% of pericardial effusion in patients suffering from systemic sclerosis (SSc)-associated pulmonary hypertension (PAH). As pericardial effusion is often associated with immunological disease even without a diagnosis of PAH, this difference is probably due to the heterogeneity of the causes and the severity of pulmonary hypertension [27].

### Primary Sjogren Syndrome (SS)

Sjogren’s syndrome is a chronic inflammatory condition associated with lymphocytic infiltration of glandular and extraglandular organs. Though numerous cytokines (IFN and IL17/IL23) and B cells play a pivotal role in the pathogenesis of SS, their role in associated pericarditis is not well defined [28].

### Vasculitis

Pericardial involvement is rare when compared to other cardiac manifestations (myocarditis, coronary disease, aortic disease) in small-, mid- and large-vessel vasculitis [29]. Although the exact pathway of pericardial inflammation is still unclear, the similar response of both pericarditis and vasculitis proves a common pathogenesis [30, 31]. The chronic exposure of vasculitis patients to immunosuppressive medications could also increase the risk of exposure to viral infections, which could in turn lead to acute or recurrent pericarditis [31].

### Clinical Presentation (Fig. 2; Table 1)

**Fig. 2:**
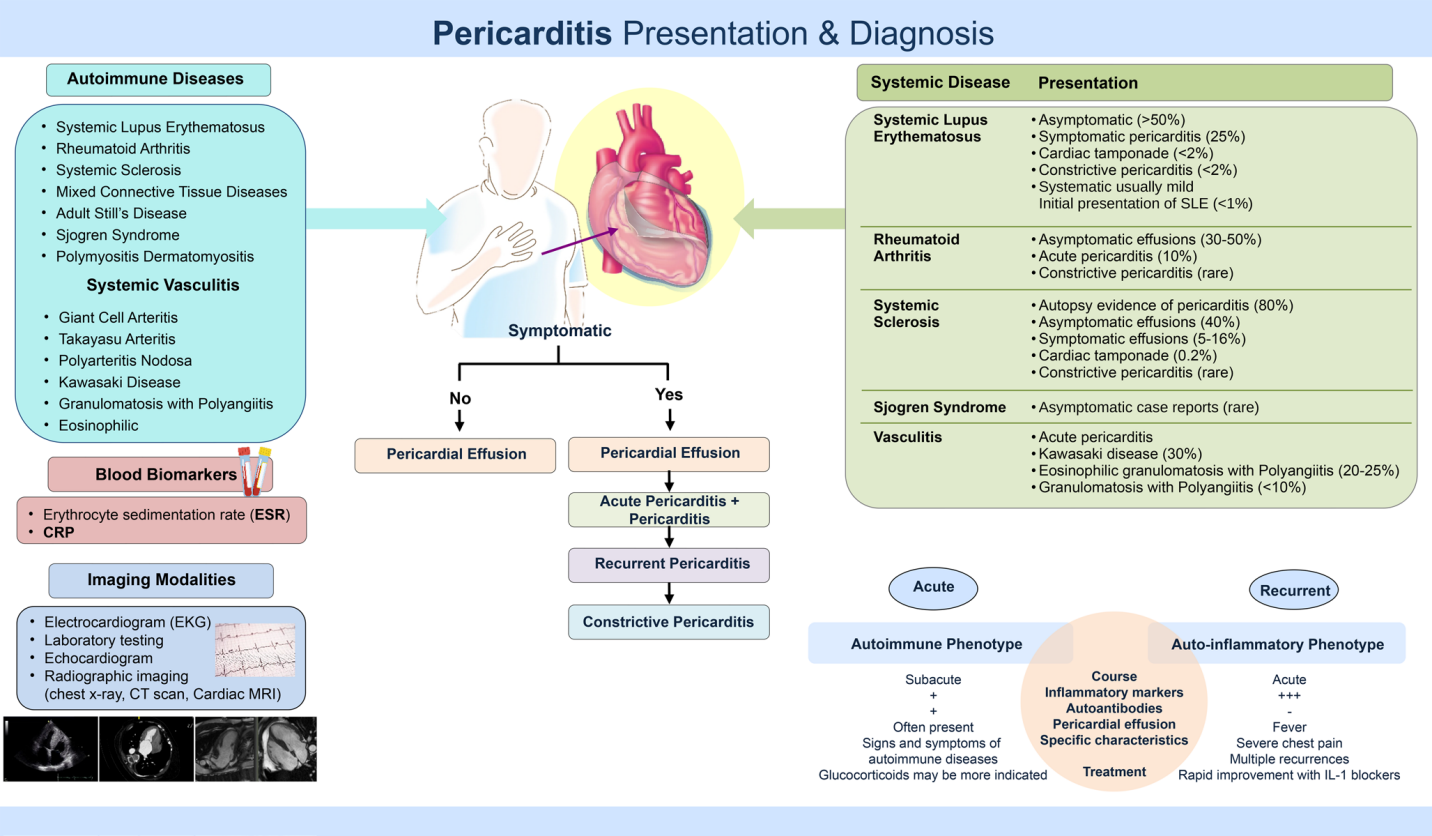
Pericarditis presentation and diagnosis

The clinical presentation of AAP in general is associated with acute inflammatory response and rarely is the initial presenting feature of the disease [32]. A recent study of 471 patients, of which 7% were noted to have autoimmune disease, described in detail the variation of presentation in patients under the age of 75 years compared to over 75 years (geriatric group). Younger patients presented more frequently with chest pain, pericardial rubs, widespread ST- segment elevation, while the geriatric group depicted more commonly dyspnea, pericardial and pleural effusion [8].

### SLE and Pericarditis

Pericarditis affects up to 50% of patients and is usually mild and rarely compromises the patients hemodynamically [22]. Pericarditis can present at onset of SLE or as part of a recurrent flare or isolated [33, 34]. There are few case reports where the initial presentation of acute pericarditis precedes the diagnosis in < 1% of patients [35, 36]. Even so, it is important to emphasize that most of the pericardial involvement in SLE is asymptomatic [37]. Factors associated with increased risk of pericardial involvement include male gender with active disease in conjunction to cytopenia, proteinuria, fever, lymphadenopathy, interstitial lung disease and Raynaud’s disease and lupus anticoagulant [38, 39].

### Rheumatoid Arthritis and Pericarditis

Similarly to SLE, pericarditis is the most frequent cardiac manifestation in RA with nearly 40% of pericardial involvement [40]. Symptomatic acute pericarditis is infrequent in RA (10% or less), while one out of three patients would have an incidental pericardial effusion on imaging. Acute presentation is usually associated with advanced disease and elevated biomarkers [41, 42]. Rare case reports display pericardial disease preceding diagnosis of RA [43]. Other pericardial manifestations seen in newly diagnosed and long-standing RA are cholesterol pericarditis and effusive-constrictive pericarditis [44]. Cholesterol pericarditis is a rare entity with chronic, large, cholesterol-rich pericardial effusions. Although the pathophysiology of this accumulation is unclear, the sources of cholesterol are thought to be blood and associated inflammation [45].

### Systemic Sclerosis and Pericarditis

Autopsy studies have shown that ~ 70% of systemic sclerosis patients had chronic pericarditis or pericardial involvement, although only 5–20% eventually develop symptoms. Risk of pericardial disease is increased 8-fold compared to the general population and mostly related to pulmonary hypertension rather than acute pericarditis [46]. Clinically symptomatic pericardial effusions are present in only 5–16% of SSc patients and pericardial tamponade is rare (0.2%). However, hospitalized patients with cardiac tamponade had a significantly increased mortality rate of 17.7%, compared to 8.8% in patients with pericardial effusions without a tamponade physiology [47].

### Sjogren’s and Pericarditis

Pericardial involvement in Sjogren’s may often be asymptomatic and remains relatively rarely reported [48, 49]. Rare cases of either acute pericarditis or constrictive pericardial disease have been reported [48].

### Vasculitis and Pericarditis

In antineutrophil cytoplasmic autoantibody (ANCA)-associated vasculitis (AAV), pleural and pericardial involvements are well recognized in eosinophilic granulomatosis with polyangiitis (EGPA) but considered rare manifestations of the other forms of AAV [3]. Giant cell arteritis (GCA), also known as temporal arteritis, is a vasculitis of large and medium-size vessels that involves the extracranial branches of the carotid artery and is of unknown etiology affecting adults age > 50 years. Cardiac manifestation is estimated to occur in about 5% of GCA patients. Pericardial involvement is rare and range from 1 - 3% [50, 51].

### Complications

#### Recurrent Pericarditis

Recurrent pericarditis (RP) is associated with substantial morbidity. There is limited data for recurrent pericarditis in autoimmune disorders. A recent letter from a single institution reported 50% higher recurrence rates in AAP as compared to that of idiopathic pericarditis [52]. Younger age, moderate-severe late gadolinium enhancement and steroid use were associated with higher recurrence rates [52]. The timing of recurrence can be impacted by the disease entity such as in SLE, where it is likely to occur within one year of the onset of pericarditis.

#### Cardiac Tamponade

A recent nationwide Japanese cohort of 20,000 hospitalized patients with pericarditis sought to investigate the rates of cardiac tamponade in AAP. There were 170 AAP, and 5,027 acute idiopathic pericarditis (AIP) patients identified. The rate of in-hospital death was not significantly different between the AIP pericarditis and AAP groups (73 of 5,027 [1.5%] vs. 3 of 170 [1.8%]; *P* = 0.75), whereas cardiac tamponade was significantly more common in patients with AAP than in those with AIP (237 of 5,027 [4.7%] vs. 15 of 170 [8.8%]; *P* = 0.023). Among patients with AAP, SLE was the most common diagnosis associated with cardiac tamponade complications, followed by rheumatoid arthritis and systemic sclerosis [53].

#### Constrictive Pericarditis

Neither the rates of constrictive pericarditis in autoimmune disorders nor the role of immunity and immunosuppressive agents in mitigating this risk are known. Further research is needed to address these gaps.

### Diagnostic Studies

#### Electrocardiogram (EKG)

Classic EKG findings include ST elevation and PR depression seen in 60% of patients with acute pericarditis, while up to 40% of patients present with atypical and non-diagnostic EKG findings [54].

#### Laboratory Data

Elevated biomarkers i.e., C-reactive protein (CRP) and estimated sedimentation rate (ESR) are the hallmark of diagnosis which may be associated with autoimmune disease activity. Pericardial fluid analysis may provide additional diagnosis value. For example, glucose level (< 45 mg/dL), elevated protein (> 5 g/dL) and leucocyte count (> 15,000/πL) may suggest RA as the underlying diagnosis. Other associated findings suggestive of RA are high pericardial RF and immunoglobulin G levels, with a low total serum hemolytic complement. In RA with nodules, cholesterol levels are seen to be elevated in the pericardial fluid. Therefore, if autoimmunity is suspected a detailed analysis of pericardial fluid may be key. In a cohort of 422 patients with recurrent pericarditis, (15% with autoimmune disease), there was a correlation of inflammatory markers such as ESR and hs-CRP (high sensitive CRP) with late gadolinium enhancement (LGE) on Cardiac MRI (CMR), which underscores that biomarkers maintain an important role in autoimmune pericarditis [55].

### Imaging Modalities

#### Chest X-ray

Plain chest radiographs (CXR) will often be normal in patients with acute pericarditis. Enlargement of the cardiac silhouette (cardiothoracic ratio (CTR) > 50%) on CXR has a moderate sensitivity (71%), but low specificity (41%). Logically, specificity increases as cardiomegaly increases (76% with CTR of 60%) [56]. In a study of 135 patients with confirmed constrictive pericarditis, pericardial calcification on CXR was present in 27% of cases. Thus, pericardial calcification is suggestive of constrictive pericarditis [57].

#### Echocardiography

Transthoracic echocardiography (TTE) remains the first line in diagnosis imaging of pericardial disease due to its widespread availability and low cost. Echo evaluation is key to determine the size of the effusion as well as the hallmarks of constrictive physiology. The size of the effusion is determined by the largest dimension of the echo-free space at end-diastole and stratified as such: trivial (only seen in systole), small < 1 cm, moderate 1–2 cm, large > 2 cm and very large > 2.5 cm [58, 59]. Transudative effusion compared to exudative effusions are determined by typical anechoic nature of the former compared to the heterogenous appearance of the later. Epicardial adipose tissue is increased in patients with autoimmune disorders [60] and may mimic pericardial effusion. Pericardial thickening is sometimes also seen in pericarditis.

Constrictive physiology involves two unique features:1) dissociation between intrathoracic and intracardiac pressure with respiration; and 2) interventricular dependance with increased diastolic filling pressure in both right- and left-sided chambers. This dynamic is translated to TTE parameters proposed as the Mayo Clinic Echocardiographic Diagnostic criteria for constrictive pericarditis, which include: a) E predominant mitral inflow with respiratory variation (E/A ratio > 0.8); b) dilated inferior vena cava usually with minimal collapse; c) Respiro-phasic ventricular septal shift caused by ventricular interdependence; d) elevated medial mitral early diastolic velocities (e’) > 8 cm/sec, often higher than compared to lateral e’ (also known as annulus reversus) and e) expiratory end diastolic hepatic venous flow reversal velocity/forward flow velocity > 0.8. The combination of respire-phasic ventricular shift and elevated medial e’ have a positive predictive value of 99% [58, 61]. Recent advances in 3D and strain suggest a role for this modality in the diagnosis of constrictive pericardial disease. Circumferential strain is more likely to be reduced in constriction compared to global longitudinal strain seen in autoimmune condition, such as SLE [62]. There is also a unique regional longitudinal strain pattern that has been demonstrated in constriction, in which the lateral ventricular strain is reduced as compared with the septal ventricular strain (also known as strain reverses). This is due to the tethering effects of the constrictive pericardium [63].

#### Cardiac CT

Cardiac CT has a supplementary and more limited role in the evaluation of pericardial disease. Pericardial thickening may be seen in pericarditis although chronicity cannot be determined, whereas pericardial calcifications can be seen in chronic constrictive pericarditis. Furthermore, cardiac CT can be used to assess pericardial effusion size and characterization can be assessed. CT also has an important role in the pre-operative planning for procedures such as pericardiocentesis and pericardiectomy [64].

#### Cardiac Magnetic Resonance (CMR)

CMR offers a comprehensive second-line modality and has increasingly rendered its value in the diagnosis and management of pericardial disease. Current CMR protocols include steady-state free precession to evaluate chamber size and function, real-time free-breathing imaging critical for assessing respire-phasic septal shift, black blood spin echo sequence with and without contrast (T1 and T2 weighted inversion-recovery turbo spin echo sequences) to elicit pericardial anatomy and thickening, T2 short tau inversion recovery (STIR) to identify pericardial edema and late gadolinium enhancement (LGE) to identify pericardial inflammation and fibrosis [58, 65].

In the acute phase, there is typically increased signal in both T2-STIR and LGE. In the chronic phase, the former disappears, and the latter is correlated with severity and outcomes. In the recent protocol predefined substudy of the RHAPSODY trial, patients with moderate-severe LGE had a higher number of recurrences than patients with mild pericardial LGE [66]. Although CMR has cemented its role in management and prognosis of pericarditis, its widespread use is hindered by cost and institutional constraints.

#### ^18^F-fluorodeoxyglucose-positron Emission Tomography/computed Tomography (^18^FDG-PET/CT)

In past decades, the role of ^18^FDG-PET/CT has grown in identifying sources of inflammation, yet its role in pericardial disease remains unclear. In 2015, European Society of Cardiology guidelines recommended ^18^FDG-PET/CT in limited cases for the diagnosis of autoimmune (large-vessel arteritis) and autoinflammatory (sarcoidosis) conditions. For example, increased FDG uptake in multiple large vessels throughout the body may indicate a disease such as large-vessel vasculitis [67]. This modality can aid in the differential diagnosis of alternative etiologies for pericarditis. In one study of 107 patients, a maximum standardized uptake value (SUV max) ≥ 5 typically was associated with tuberculosis or neoplastic or autoimmune disease compared to < 5 in idiopathic pericarditis [68]. One of the major limitations of ^18^FDG-PET/CT in autoimmune disorders is the reduced sensitivity with the use of corticosteroids for diagnosing inflammation. This has been demonstrated in several diseases, including large-vessel vasculitis, rheumatoid arthritis, and polymyalgia rheumatic [69]. As such, it is recommended to delay the commencement of corticosteroid treatment until FDG-PET/CT is performed, unless there is a risk of severe complications such as ocular ischemia in temporal arteritis. ^18^FDG-PET/CT is an adjuvant tool where the etiology of pericarditis is unclear.

### Treatment (Table 2)

**Table 2:** Medications used in autoimmune pericarditis (administration, side effects, and efficacy)

Medication	Administration	Side Effects	Efficacy	Notes
Aspirin/ NSAIDs	Aspirin: oral 650-1000 mg TID for 1–2 weeks, then decrease 250mcg per week after resolution. Ibuprofen: oral 600-800 mg TID for 1–2 weeks, then decrease 200 mg/week Indomethacin: oral 25-50 mg TID for three months (acute) or 6 + months (recurrent) without taper.	Primarily GI (gastritis, ulcers), bleeding risk.	Rapid response predicts favorable prognosis in acute pericarditis. If pain/fever persist > 1 week or effusion develops, suspect autoimmune or other cause.	First line. Mainstay for idiopathic and viral. Limited by renal dysfunction in lupus nephritis.
Colchicine	Weight-based dosing: < 70 kg: 0.5-0.6 mg daily ≥ 70 kg: 0.5-0.6 mg BID Given for three months (acute) or 6 months (recurrent).	Well tolerated. Diarrhea, nausea, vomiting. Rarely transaminitis, alopecia, neuropathy.	Reduces rates of incessant/recurrent pericarditis and improves remission by 30%.	First line. 80% hepatic metabolism. Inhibits microtubule assembly interfering with innate immune response
Steroids	Prednisone, oral, 0.2-0.5 mg/kg daily for 2–4 weeks (acute or recurrent). Gradual taper over 2–3 months.	Typical steroid side effects based on dose and population.	Long-term use increases risk of recurrence in idiopathic AP and RP but risk of recurrence is unknown in autoimmune pericarditis	First line. Widely used for disease management of autoimmune disorders that may underlie pericarditis
Azathioprine	Initial dose 1 mg/kg daily, gradually increased to 2-3 mg/kg taken for many months (max dose 150 mg daily).	Well tolerated. Transient hepatotoxicity, leukopenia, transient GI symptoms. Avoid concomitant therapy with allopurinol, which increases bone marrow toxicity.	Slow onset of action (1–2 months) requires therapy for several months. Associated with stable remission after steroid discontinuation in > 50% patients.	Fourth line. Purine metabolism antagonist used off-label for idiopathic recurrent pericarditis. Not for acute attacks.
Anakinra	1-2 mg/kg subQ daily (max dose 100 mg daily) for several months with slow taper over three months.	Very rarely opportunistic infections (M. tuberculosis). Rarely neutropenia, transaminitis, arthralgia. Screen for HBV, tuberculosis. Contraindicated if active infection or pre-existing malignancy.	Reduces risk of recurrence in patients with recurrent pericarditis over months. Limited evidence on withdrawal protocols—likely should gradually taper based on clinical evidence of remission.	Fourth line. IL-1 receptor antagonist (both IL-1α and IL-1β) used in many inflammatory diseases. Used as a single agent or with colchicine.
Canakinumab	Administer every 4–8 weeks. 150 mg/4 weeks subQ for adults. No hepatic or renal dose adjustments.	Rarely increased uric acid (gout flare), diarrhea, neutropenia, headache.	Approved for use in autoinflammatory diseases where pericarditis is a component particularly periodic fever syndromes (PFS) and Stills disease but use for isolated pericarditis is limited to case reports. No use in autoimmune pericarditis	Fourth line. Monoclonal antibody against IL-beta. Longer half-life than Anakinra (22–26 days). Selectively inhibits function of IL-1β.
Rilonacept	Fixed dose of 160 mg subQ weekly after a loading dose of 320 mg subQ. No dose adjustment for hepatic/renal impairment.	Rarely infections, antibody development.	RHAPSODY Trial showed efficacy in recurrent pericarditis. Autoimmune pericarditis was excluded. Case reports use.	Fourth line. Blocks IL-1α and IL-1β.
Goflikicept	Half-life is longer than Rilonacept allowing less frequent dosing: once every two weeks.	Rarely, hypercholesterolemia, infections.	An option after failure of NSAIDs/colchicine and before glucocorticoids. In phase III trial. Role is unknown in autoimmune pericarditis	Fourth line. IL-1 inhibition, structurally different from Rilonacept (smaller molecular mass).

Acute and recurrent pericarditis has been reported in association with treatments such as corticosteroids, hydroxychloroquine, mycophenolic acid, cyclophosphamide, and azathioprine. As a result, a standard protocol previously suggested for idiopathic acute and recurrent pericarditis may be of limited utility [39]. The understanding and knowledge of available therapies is critical to tailor therapies effectively. First-line treatment for acute pericarditis includes non-steroidal anti-inflammatory drugs (NSAIDs) such as ibuprofen or aspirin, combined with colchicine. Corticosteroids are considered as well depending on the severity of presentation, particularly for patients who do not respond to NSAIDs and colchicine or have contraindications to these medications. For refractory cases or those with multiple recurrences, interleukin-1 (IL-1) inhibitors such as anakinra and rilonacept have shown efficacy. Immunosuppressive agents like azathioprine and intravenous immunoglobulins (IVIG) may be considered in severe, refractory cases, when corticosteroids and IL-1 inhibitors are insufficient.

In summary, the treatment of acute and recurrent autoimmune pericarditis involves a stepwise approach starting with NSAIDs and colchicine, progressing to corticosteroids if necessary, and considering IL-1 inhibitors and immunosuppressive agents for refractory cases.

### Conventional Anti-Inflammatory Therapies

#### Aspirin and NSAIDs

Aspirin and NSAIDs are the mainstay of medical therapy for acute and recurrent idiopathic pericarditis. A rapid response to aspirin or other NSAID therapy predicts a favorable prognosis in acute pericarditis. However, in the absence of previously diagnosed autoimmune disease, if chest discomfort or fever persists > 1 week, or a new or larger pericardial effusion develops during therapy, a cause of pericarditis other than post viral/idiopathic should be suspected. In a report of 254 patients with acute pericarditis treated as outpatients with aspirin, 61% of those with symptoms who did not respond by 7 days of therapy were found not to have idiopathic pericarditis. 43% were determined to have autoimmune conditions, and 18% had tuberculosis [70]. The recommended dosing of Aspirin (750-1,000 mg) and NSAIDS (Ibuprofen, 600–800 mg; Indomethacin 25-50 mg) every 8 h for 1–2 weeks may be detrimental to patients with autoimmune disorders with renal involvement, limiting its utility as a first-line drug [54].

#### Colchicine

Since its introduction as an adjuvant therapy to conventional anti-inflammatory therapies in 2013, colchicine has become a mainstay in medical management of pericarditis. Colchicine not only reduces the rates of incessant or recurrent pericarditis but also improves remission rates by 30% [71]. However, few patients with autoimmune conditions were included in this seminal study. Colchicine’s mechanism of action is related to the inhibition of microtubule assembly interfering with key functions of the innate immune response such as inflammasome formation, cytokine secretion, and neutrophil migration. Colchicine may be safe and effective in treating SLE pericarditis and used as a steroids-sparing agent. These preliminary results need to be confirmed in a larger study with a longer follow-up [72]. Colchicine is dosed based on weight, such that patients weighing less than 70 kg should receive colchicine 0.5 mg to 0.6 mg once daily and those weighing greater than or equal to 70 kg should receive colchicine 0.5 mg to 0.6 mg twice daily. It is typically continued for 3 months and is well tolerated. The most common side effects include gastrointestinal disturbances such as diarrhea, nausea, or vomiting leading to early discontinuation in approximately 10% of cases [73].

#### Corticosteroids

Corticosteroids are traditionally the second-line agent used in pericarditis if patients’ symptoms persist or recur on the first-line dual therapies above. Although also widely used in patients with autoimmune disorders, long-term use of corticosteroids for treatment of pericarditis increases the risk of recurrence, in particular at high doses  > 20 mg daily and should not be the mainstay of therapy even if used for disease management [74].

### Advanced Anti-Inflammatory Therapies

#### Azathioprine

Azathioprine (AZA), a purine metabolism antagonist, has been approved by the Food and Drug Administration (FDA) for the symptomatic treatment of active rheumatoid arthritis. It also has approval as an adjunctive therapy for the prevention of kidney transplant rejection. In addition, it is used off-label for a multiple other autoimmune conditions such as inflammatory bowel disease, multiple sclerosis and dermatomyositis just to name a few [75]. AZA has a slow onset of action (1–2 months) that necessitates prolonged therapy of several months. Therefore, it is not useful in the resolution of an acute attack but rather as a long term agent [76]. AZA has been suggested for treatment of pericarditis associated with SLE, RA, systemic sclerosis, Sjogren and Vasculitis [4, 77]. The recommended initial dosage is 1 mg/kg once daily, gradually increased to 2–3 mg/kg per day. Concomitant therapy with allopurinol greatly increases bone marrow toxicity and must be avoided [78]. With the advent of IL-1 inhibitors, AZA has fallen out of favor as an agent in the management of recurrent or incessant pericarditis.

#### Anakinra

Anakinra is a recombinant human interleukin-1 (IL-1) receptor antagonist that blocks both IL-1 alpha and IL-1 beta to prevent inflammation. Therefore, it can potentially be used to treat a multitude of inflammatory diseases, such as RA and cryopyrin-associated periodic syndromes.

In a randomized clinical trial, AIRTRIP, Anakinra reduced the risk of recurrence over a median of 14 months in patients with recurrent pericarditis compared to colchicine resistance and corticosteroid-dependent patients [79]. Furthermore, analysis of a registry data of patients (11% with autoimmune and autoinflammatory 9% and 2% respectively) who were steroid- and colchicine-dependent, indicated a 6-fold reduction in recurrences and an 11-fold reduction -in emergency department admissions and hospitalization after treatment with Anakinra over a 6-month period [80]. The recommended daily dose is 100 mg subcutaneously adjusted to every other day if GFR < 30 mg/dl.

Although Anakinra is considered a safe agent, without significant risk of opportunist infection compared to other biologics, the high cost and the long duration of therapy before withdrawal of conventional treatment have precluded its widespread use [81].

Anakinra has been approved for the treatment of RA. The administration of anakinra in combination with other anti-inflammatory drugs improves symptoms and reduces levels of inflammatory biomarkers. Preliminary data from a single center study of 31 patients, with autoimmune disorders, primarily with SLE (40%) who were refractory to treatment with disease-modifying agents aimed at the underlying disorder, and who were treated additionally with Anakinra or Rilonacept, had a decrease in number of flares. There was no evidence of significant adverse events, which suggests a relatively safe profile of the use of IL -1 inhibitors in the setting of other disease-modifying agents in select cases [82].

#### Canakinumab

Canakinumab is a human monoclonal antibody directed against IL-beta. Compared with Anakinra, it has a longer half-life that ranges from 22 to 26 days, which means that it can be administered every 4 to 8 weeks. The dosage used on adults is 150 mg/4 weeks by subcutaneous injection. There is no hepatic or renal dose adjustment required [83]. Its current FDA-approved indications are cryopyrin-associated periodic syndromes (CAPS), familial Mediterranean fever (FMF), tumor necrosis factor-alpha associated period syndrome (TRAPS), hyper-immunoglobulin D syndrome/mevalonate kinase deficiency (HIDS/MVK), adult-onset Stills disease, systemic juvenile idiopathic arthritis (JIA). Pericarditis is a component of many of these disorders, yet the use of Canakinumab for isolated pericarditis is limited to case reports [84]. Unlike Rilonacept and Anakinra which target both IL-1α and IL-1β, Canakinumab selectively inhibits the function of IL-1β. This may potentially explain its limited efficacy is the management of isolated pericarditis not associated with autoinflammation. To summarize, there is no role for canakinumab in autoimmune conditions.

### Rilonacept

Rilonacept is a homodimer fusion protein consisting of the two ligand binding domains of both IL-R (IL-1-R1) and the accessory IL-1 receptor protein((IL-1-RAcP), linked in-line to the FC portion of human IgG1 that blocks the activities of both IL-1α and IL-1β [85]. It is prescribed at a fixed dose of 160 mg SQ weekly after a loading dose of 320 mg SQ. Hepatic and renal impairment have no impact on the drug's pharmacokinetics, thus no dose adjustment is required. The seminal clinical randomized trial “RHAPSODY” provided supportive data for the efficacy and safety of Rilonacept in RP. Among the 86 patients included in the study, autoimmune pericarditis was excluded [86]. In a recent case series of patients with lupus pericarditis, Rilonacept was used successfully in four patients with SLE with resolution of symptoms in 3 out of 4 patients after failed treatment regimen the included hydroxychloroquine, methotrexate, steroid, colchicine in addition to rituximab, belimumab and anakinra. Concomitant medications were not clear. This suggested that in a subset of patients with SLE, IL-1 is the main mediator of disease activity and would benefit from IL-1 inhibition therapy [87]. Further studies are necessary to evaluate rilonacept’s use in autoimmune pericarditis along with optimal duration of therapy and weaning strategies.

### Goflikicept

Goflikicept is a new fusion protein, with IL-1 inhibition properties, while structurally different from Rilonacept. Goflikicept is a heterodimeric protein in which the 1 IL-1-R1 domain is placed on 1 polypeptide chain of the molecule while 1 IL-1-RAcP domain is placed on another polypeptide chain. Thus, Goflikicept has a significantly smaller molecular mass leading to a simpler and less expensive manufacturing process. The half-life of Goflikicept is longer than that of Rilonacept allowing less frequent (once every 2 weeks) administration [88].

In patients with auto-immune conditions who are receiving immunosuppressive therapy of variable efficacy and strength and who experience acute and recurrent pericarditis, the long-term safety of the addition of IL-1 inhibitors remains unclear. The main challenge is that over 100 types of autoimmune disorders and a growing number of biologic agents have been identified. There are neither randomized nor substantial observational studies that would determine the safety profile and outcomes. There have been proposed tiered system where the use of IL-1 may have limited increase in risk of infection with conventional synthetic DMARDS (Methotrexate, sulfasalazine, leflunomide, hydroxychloroquine) compared to biologic (TNF inhibition, Anti CD20 Rituximab, IL-6 inhibition Tocilizumab, CD80/CD86 Inhibition Abatacept) and targeted DMARDS (Jak inhibitors).

### Surgical Management

In the case of recurrent autoimmune pericarditis with persistent symptoms despite trials of the multiple medical regimens described above, surgical pericardiectomy can be considered. Pericardiectomy is an open-heart surgical procedure where the constricting parietal and epicardial layers of pericardium are dissected away [2]. While shown to be effective and safe when done at major cardiac centers, pericardiectomy is typically reserved for chronic constrictive pericarditis and less common medically refractory inflammatory pericarditis. With respect to recurrent pericarditis broadly, the 2015 European Society of Cardiology (ESC) guidelines recommend pericardiectomy only as a last resort for patients with pericarditis refractory to all medical therapies. However, newer evidence suggests that earlier surgical intervention is associated with better outcomes from a symptom perspective [89].

Most of the data on pericardiectomies examine all cases of constrictive pericarditis together and include autoimmune pericarditis in the category of idiopathic pericarditis. While idiopathic pericarditis often makes up the bulk of cases, more work is needed examining outcomes from pericardiectomies specifically in the autoimmune pericarditis population. Pericardiectomy is indicated in patients with constrictive pericarditis with moderate to severe symptoms that have persisted despite 4–12 months of IL-1 inhibitor therapy, who have not progressed to end-stage disease and do not have significant high-risk comorbidities. The procedure is typically avoided in patients with mixed constrictive-restrictive disease, such as restrictive cardiomyopathy from radiation on top of autoimmune disease, as they have worse outcomes [90]. Complete pericardiectomy is preferred over a partial pericardiectomy due to better outcomes, as well as high operative mortality associated with repeat pericardiectomies [91].

The benefits of a successful surgical pericardiectomy are also significant. Patients are spared months to years of immunosuppressive therapy and experience symptomatic relief.

### Future Perspectives

The vast heterogeneity of the pathogenesis, presentation and prognosis of autoimmune disorders renders itself to the development of precision medicine. Integration of big data sets of well-defined cohorts of autoimmune patients will provide a granular understanding of disease mechanisms and subsequent tailoring therapies. A recent meta-analysis of genome-wide association (GWAS) studies (including 4894 pericarditis patients, from 5 countries) identified 2 independent common intergenic variations at the interleukin 1 (IL-1) locus on chromosome 2q14. The lead variant was associated with lower rates of recurrent pericarditis suggesting a particular importance of the locus in the pathogenesis of this persistent disease subset [92]. This study heralds a new age in which genetic evaluation of pericarditis may alter prognosis and therapy. Furthermore, development of risk stratification models to predict long-term outcomes of “clinical remission” in patients with recurrent pericarditis and those at risk of recalcitrant pericarditis utilizing machine learning is another step in personalized treatment and follow-up in such a heterogenous disease [93]. An unsupervised model integrating transcriptome and methylome data of patients with systemic autoimmune disorders observed 3 pathologic clusters that were described as inflammatory, lymphoid, and interferon clusters. This reclassification and paradigm shift has implications for future clinical trials and the study of nonresponse to therapy, in systemic autoimmune diseases [15]. Future studies in pericardial disease specifically are needed to predict response to therapy, in addition to use of multiple biologic agents and their safety profile.

## Conclusions

Autoimmune pericarditis is a unique phenotype of pericarditis that warrants further research. There is scarce data of the pathophysiology of acute and recurrent pericarditis in autoimmune disorders. Since subtypes may benefit from IL-1 inhibition, future phenotyping based on clinical data, biomarkers and imaging is prime to determine future available therapies as the field for therapies expands. Further research to understand the mechanisms of pericarditis in each disease entity will provide deeper insight into the use of IL-1 inhibitors in these patients.

## Key References

- Klein AL, Wang TKM, Cremer PC, Abbate A, Adler Y, Asher C, et al. Pericardial Diseases: International Position Statement on New Concepts and Advances in Multimodality Cardiac Imaging. JACC Cardiovasc Imaging. 2024;17 [8]:937 − 88.
-
  - *This article highlights the importance of new cardiac imaging modalities in diagnosing pericarditis and guiding management.*
- Yesilyaprak A, Kumar AK, Agrawal A, Furqan MM, Verma BR, Syed AB, et al. Predicting Long-Term Clinical Outcomes of Patients With Recurrent Pericarditis. J Am Coll Cardiol. 2024;84 [13]:1193 − 204.
-
  - *This article includes all the clinical*,* laboratory and imaging characteristics of patients with recurrent pericarditis and provides a prediction score for risk of recurrence*,* which helps in setting expectations with patients and guiding management.*

## Data Availability

No datasets were generated or analysed during the current study.
